# A PIG Localization Method Based on Distributed Optical Fiber Vibration Sensing and Image Matching

**DOI:** 10.3390/s26216816

**Published:** 2026-10-28

**Authors:** Yunbin Ma, Jinbo Chen, Yingchao Fang, Ailing Wang, Haiming Wang, Mingzhu Xue, Wenghang Xu, Yatao Pan, Shuaiqi Liu, Yongkang Dong, Taofei Jiang

**Affiliations:** 1 School of Software Engineering, Xi’an Jiaotong University, Xi’an 710049, China; 2 PipeChina Institute of Science and Technology, Tianjin 300457, China; 3 National Key Laboratory of Laser Spatial Information, Harbin Institute of Technology, Harbin 150001, China; 4 Zhengzhou Advanced Research Institute of Harbin Institute of Technology, Zhengzhou 450007, China; 5 Southwest Pipeline Company Limited, PipeChina Company, Chengdu 610218, China

**Keywords:** distributed vibration sensing, pipeline inspection gauge, real-time localization, dark fiber

## Abstract

In-line inspection of long-distance oil and gas pipelines is a critical maintenance measure to ensure pipeline structural integrity and the safe, efficient operation of transportation systems. Nevertheless, the Pipeline Inspection Gauge (PIG) is inevitably at risk of jamming while travelling through pipelines. Therefore, accurate PIG localization is essential for securing in-line inspection operations. Conventional PIG detection techniques fail to achieve full-range continuous positioning and monitoring of PIGs, exhibiting notable technical limitations. To address this issue, this paper proposes a spatiotemporal PIG localization method that combines Distributed Vibration Sensing (DVS) technology with a cross-correlation template-matching algorithm. The innovations of the proposed method are summarized as follows: (1) A DVS system is established. Sliding cross-correlation matching is performed by introducing a standard signal template into the time-frequency waterfall plot to accurately extract spatiotemporal position information of the moving PIG. (2) A physics-driven, training-free localization template is proposed to reduce dependence on labeled data and facilitate deployment across different pipeline sections. (3) The method requires no model training and enables weld-impact event localization through lightweight signal processing. Field experiments using a 40-km DVS system demonstrate its feasibility in the observed pipeline sections. Field experiments using a 40-km DVS system demonstrate the feasibility of identifying and localizing PIG-induced weld-impact events within the experimentally observed pipeline sections.

## 1. Introduction

As the lifelines of energy transportation, the safe operation of oil and gas pipelines is critical to national energy security and economic stability [1,2]. However, over long-term service, pipelines inevitably suffer from the accumulation of corrosion products and sediment deposition, which not only reduce transmission efficiency but also increase the risk of localized corrosion and safety incidents [3,4,5]. Regular pipeline cleaning and inspection runs have therefore become an essential measure to maintain pipeline safety and efficiency. Pipeline inspection gauges (PIGs) are driven by the pressure differential across them and remove internal contaminants through the interference fit between their rubber cups and the pipe wall [6]. Nevertheless, long-distance pipelines often extend for tens or even hundreds of kilometers across complex terrain, where welds, elbows, deformations, and internal deposits can all cause a PIG to become stuck [7]. If a stuck PIG cannot be promptly located and freed, the resulting shutdown will lead to significant economic losses; in severe cases, localized pressure buildup may even cause pipeline rupture [8]. Consequently, real-time, continuous tracking of the PIG during operation is a fundamental prerequisite for the successful completion of pipeline cleaning and inspection tasks and for mitigating associated safety risks.

Traditional PIG positioning methods primarily include the ground marker method and the electromagnetic pulse method [9]. The ground marker method uses low-frequency magnetic receivers pre-installed along the pipeline; when the PIG passes directly beneath a marker, it triggers a recording and thus provides point-based positioning [10]. However, this method yields only discrete position data at known marker points, leaving the entire trajectory between adjacent markers as a monitoring blind spot. Positioning accuracy depends on the density of marker deployment; increasing the number of markers can improve accuracy but substantially raises project costs and may even become impractical. The electromagnetic pulse method requires monitoring personnel to carry receiving equipment and track the PIG’s signal along the pipeline’s surface. This approach is labor-intensive, heavily constrained by terrain, and limited in effective signal reception range. In essence, it remains a point-to-point tracking method, making it difficult to achieve continuous, real-time positioning over the entire route. In recent years, alternative methods based on differential pressure [11], ultra-low-frequency magnetic fields [12], and pressure waves [13] have been explored to some extent; however, their applicability and real-time performance in complex terrain environments remain challenging.

The rapid development of phase-sensitive optical time-domain reflectometry (Φ-OTDR) has opened a new technical path for PIG positioning [14]. Φ-OTDR systems utilize redundant optical fibers in communication cables laid alongside the pipeline as distributed sensing media, detecting external vibrations by measuring the intensity variations in Rayleigh backscattered light as optical pulses travel along the fiber [15]. This technology offers significant advantages, including a long sensing range, immunity to electromagnetic interference, no need for external power supply, high sensitivity, and low cost [16]. As the PIG travels along the pipeline, its front and rear rubber cups continuously impact the girth welds, and the resulting pressure fluctuations propagate upstream and downstream at the acoustic velocity of the pipeline medium. These pressure fluctuations, in turn, excite vibrations in the pipe wall that propagate through the wall and surrounding soil to the optical fiber, appearing as distinctive “V”-shaped energy fringes in the spatiotemporal map captured by the DVS system. The apex of the “V” corresponds to the impact location at the weld. This physical signature provides a critical basis for visual-pattern-based PIG positioning methods.

Several studies have investigated PIG positioning using distributed fiber-optic sensing technology, but there is an urgent need to develop low-cost and reliable identification methods. Sha et al. [17] utilized Φ-OTDR signals to construct spatiotemporal maps and employed YOLOv3 for end-to-end detection of V-shaped features. Du et al. [18] combined semantic segmentation with edge detection, first using an improved U-Net network to segment and extract V-shaped regions, and then applying the Canny operator and Hough transform for feature point localization. Zhu et al. [19] designed a three-module framework consisting of denoising reconstruction, dual feature extraction, and logical inference based on time-domain features. Huang et al. [20] applied traditional image processing methods to DAS data, using clustering algorithms and the Hough transform to determine the PIG position under different operating conditions.

However, the above methods generally suffer from recognition instability due to degraded signal quality under complex operating conditions. Along long-distance pipelines, background noise stems from various sources, including vehicle traffic, construction machinery, rainfall, and other environmental disturbances. When the PIG’s impact signals are weak, the “V” pattern may exhibit local discontinuities, blurred edges, or may even be entirely submerged by background noise in the spatiotemporal map, leading to missed detections and positioning drift for deep learning models that rely on complete textural features. In particular, when certain fiber-optic segments are located far from the actual pipeline route, the received vibration signals attenuate significantly, making the “V” pattern increasingly indistinct. Under such conditions, methods that rely solely on spatial morphological texture often fail, and robustness is difficult to guarantee. Furthermore, training deep learning models requires a large volume of manually labeled data. However, signal characteristics vary considerably across different pipelines due to differences in pipe diameter, wall thickness, transported medium, and burial environment, limiting the models’ cross-pipeline generalization capability. Transferring a trained model to a new pipeline requires recollecting data and adjusting the model, which to some extent restricts the efficiency of large-scale field deployment.

**Figure 1:**
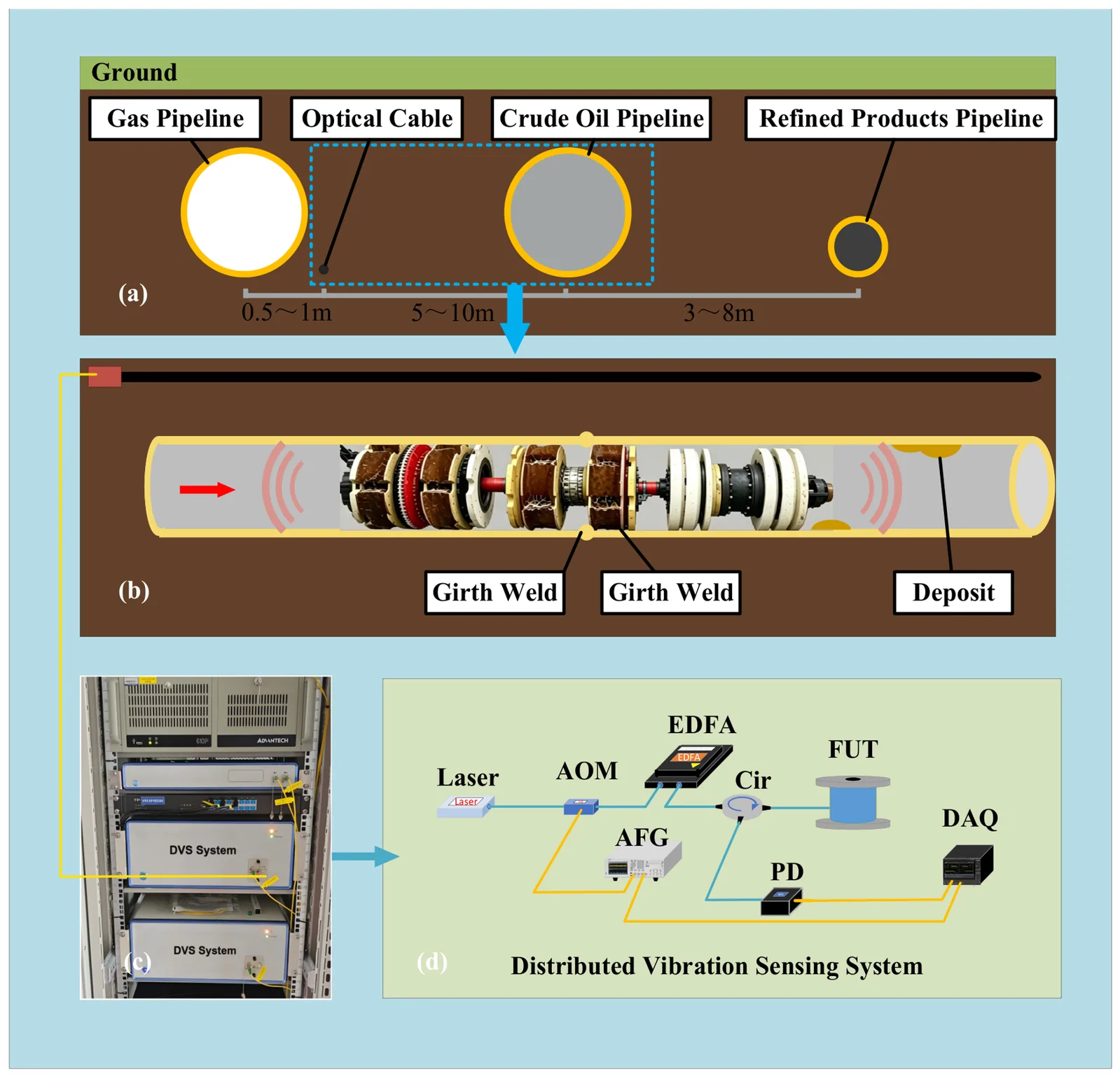
(**a**) Cross-sectional view of natural gas, crude oil, and refined oil pipelines running parallel with a fiber-optic cable. (**b**) A PIG striking a weld seam in a crude oil pipeline generates vibration signals that are captured by the accompanying fiber-optic cable. (**c**) The field-deployed DVS system accesses a redundant optical fiber in the communication cable to achieve single-ended sensing. (**d**) Schematic diagram of the direct-detection DVS system.

To address these limitations, we propose a training-free method for localizing PIG-induced weld-impact events using direct-detection DVS. Wavelet filtering and directional convolution enhance weak V-shaped signals, while a physics-driven template based on acoustic propagation and PIG motion characteristics is matched to the spatiotemporal waterfall plot through two-dimensional cross-correlation. The method is evaluated under pipeline–fiber separations of 5–10 m (Figure 1). The main contributions of this work are threefold:

I. We validate the feasibility of a low-cost direct-detection DVS system in weak-coupling scenarios. Through field experiments, we demonstrate that even when the horizontal separation between the pipeline and the accompanying fiber-optic cable reaches 5–10 m, the proposed method can still reliably recover the vibration signals induced by PIG impacts.
II. We propose a physics-driven, training-free localization template. The cross-correlation matching template is strictly constructed from intrinsic pipeline parameters (sound velocity in crude oil and PIG traveling speed), which inherently circumvents the dependence on extensive manually labeled data and the poor cross-pipeline generalization bottleneck of deep-learning-based methods, enabling plug-and-play deployment on new pipeline segments.
III. We evaluate the localization performance and accuracy of the proposed method through a 40-km field trial on a crude oil pipeline in Southwest China. Quantitative comparison with the Hough transform demonstrates higher localization accuracy for PIG-induced weld-impact events under most tested parameter settings, while parameter sensitivity analysis reveals the factors affecting localization errors.

## 2. Principle

### 2.1. Direct-Detection DVS System Principle

The direct detection scheme of the DVS system is based on phase-sensitive optical time-domain reflectometry (Φ-OTDR). Its core principle is to utilize the multi-beam interference effect of Rayleigh backscattering excited in the optical fiber by highly coherent narrow-linewidth laser pulses, thereby directly converting the phase change caused by external vibrations into intensity variations for detection. After a narrow-linewidth laser pulse is injected into the sensing fiber, the backward Rayleigh scattered light generated by numerous randomly distributed scattering points within the pulse coverage range returns to the input end simultaneously. Since the coherence length of the light source is much greater than the spatial length of the pulse, the Rayleigh backscattered fields interfere coherently, and the detected optical intensity is proportional to the squared magnitude of the superposed optical field:

(1) $$I(t)\propto {\left|\sum_{i=1}^{N}a_{i}exp[j\phi_{i}(t)]\right|}^{2}$$

where $a_{i}$ and $\phi_{i}(t)$ denote the optical field amplitude and time-dependent phase of the i-th scattering center, respectively, and N is the number of contributing scattering centers. When a section of the fiber is subjected to axial strain induced by acoustic waves or vibrations, the relative optical path difference between the scattering points at that location changes, causing the phase $\phi_{i}(t)$ to vary. This, in turn, leads to significant fluctuations in the resultant light intensity. By continuously recording the backscattering traces and analyzing the intensity variation over time at the same position, the vibration waveform can be recovered. The spatial localization of vibration events is based on the principle of optical time-domain reflectometry: if the scattered signal is received at time τ after the emission of the pulse, the distance to the event is:

(2) $$z=c\tau ∕(2n_{g})$$

where (c) is the speed of light in vacuum and ($n_{g}$) is the group refractive index of the optical fiber. The nominal spatial resolution is determined by the optical pulse duration ($T_{p}$), as expressed in Equation (3):

(3) $$∆z\approx cT_{p}∕(2n_{g})$$

The term “direct detection” refers to measuring Rayleigh backscattered light intensity directly using a photodetector, without a local oscillator or phase demodulation, thereby simplifying the system architecture. The optical system consists of a narrow-linewidth laser, an acousto-optic modulator (AOM), an erbium-doped fiber amplifier (EDFA), a circulator (Cir), a fiber under test (FUT), a photodetector (PD), and a data acquisition and processing unit (DAQ). Unlike phase-demodulated distributed acoustic sensing (DAS) systems that can provide quantitative dynamic strain measurements, the proposed DVS system detects relative optical intensity variations induced by vibrations. Consequently, the measured signal amplitude cannot be directly converted into absolute vibration amplitude. Therefore, the system primarily focuses on vibration event detection and localization through differential signal processing.

### 2.2. Principle of Template Matching Technology

Two-dimensional cross-correlation is the core operation of template matching. Let the binary image to be searched be f[x,y], with size M_f_ × N_f_, and the standard template be k[x,y], with size M_k_ × N_k_. The two-dimensional discrete cross-correlation is defined as:

(4) $$\left(f⊗k\right)\left[x,y\right]=\sum_{m=0}^{M_{k}-1}\sum_{n=0}^{N_{k}-1}f\left[x+m,y+n\right]\cdot k\left[m,n\right]$$

Compared with cross-correlation, the convolution kernel’s indices are taken in the negative direction, which is equivalent to first rotating the kernel 180° around its center and then performing sliding multiplication. If the flipped kernel is defined as:

(5) $$k_{flip}[m,n]=k[-m,-n]$$

Then, there exists an equivalence relationship between convolution and cross-correlation:

(6) $$f⊗k=f*flip\left(k\right)$$

**Figure 2:**
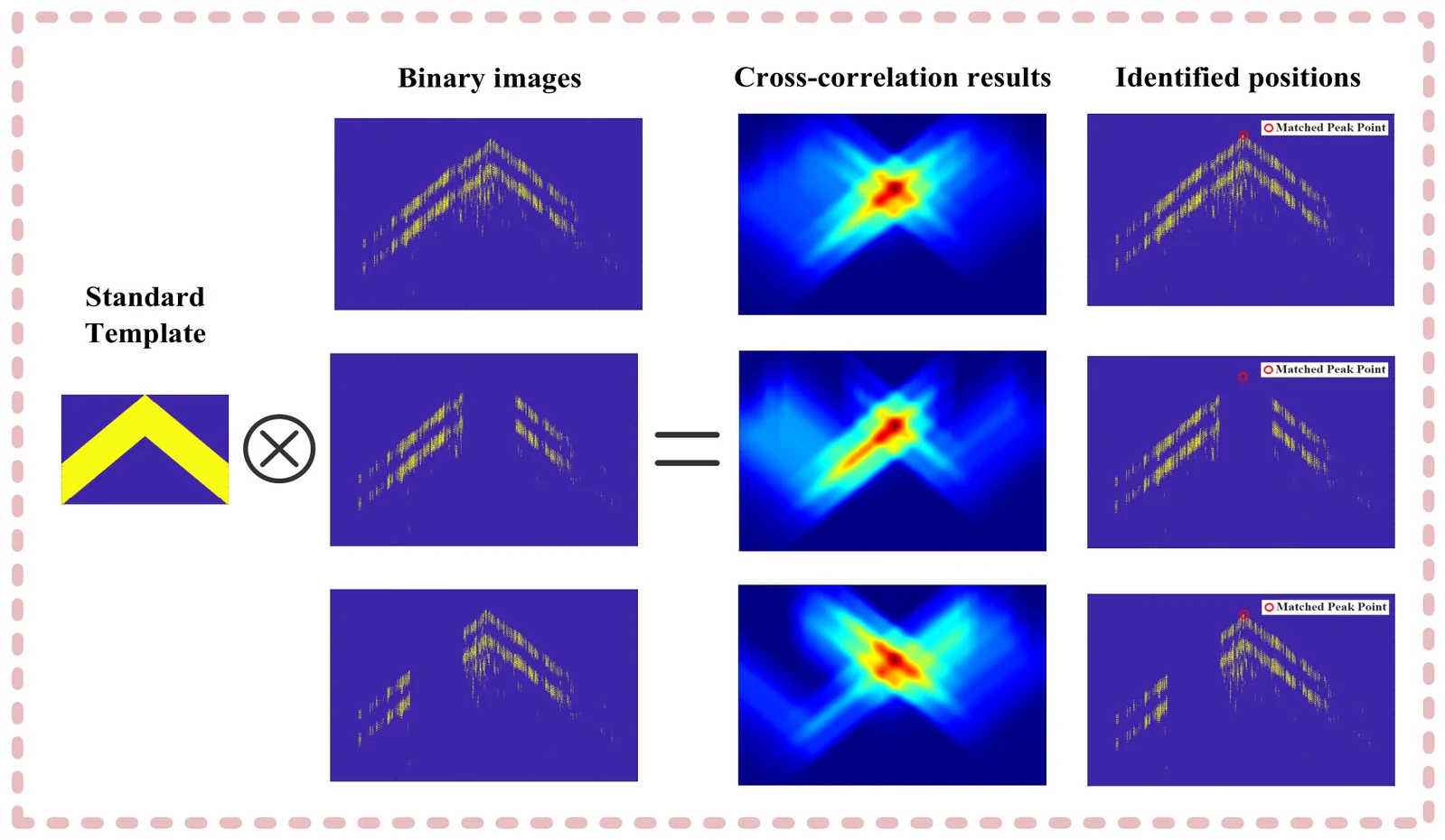
Cross-correlation results between the standard template and binarized images under multiple conditions. The maximum point represents the optimal matching center of the template, from which the location of the impact point can be determined.

In PIG localization and tracking, the standard template is cross-correlated with the processed binarized waterfall plot to find the best matching point, thereby accurately locating the weld seam struck by the PIG. Even in areas such as tunnels, bridges, and crossings where the pipeline and fiber-optic cable are separated and signal loss occurs, this method can still achieve localization and identification (Figure 2).

In this study, localization refers to identifying the positions and occurrence times of PIG-induced weld-impact events along the sensing fiber, rather than continuously estimating the physical position of the PIG between welds.

The two-dimensional cross-correlation in Equation (4) can be efficiently implemented via fast Fourier transform (FFT), reducing the overall computational complexity to O(PQlog(PQ)), which is critical for real-time tracking of long-duration waterfall plots.

The V-shaped matching template is constructed based on the acoustic propagation velocity and PIG traveling speed. When the PIG strikes a weld, acoustic waves propagate upstream and downstream, forming two inclined branches in the spatiotemporal waterfall plot. The propagation characteristics can be approximated by $t=t_{0}+|x-x_{0}|/v_{a}$, where $v_{a}$ is the acoustic propagation velocity, and $\left(x_{0},t_{0}\right)$ represents the impact event. The temporal extent of the template is estimated as $T=L_{c}/v_{p}$, where $L_{c}$ is the axial length of the sealing cup and $v_{p}$ is the PIG traveling speed.

For the field experiment, the measured acoustic velocity in crude oil was approximately 1092 m/s, and the PIG speed was approximately 1 m/s. These parameters, together with the spatial and temporal sampling intervals, were used to determine the template geometry. The resulting template dimensions were 655 × 5560 pixels, with an arm thickness of 80 pixels. The template was then matched with the binarized waterfall plot using two-dimensional cross-correlation, and the maximum correlation response was used to identify the weld-impact event position. The influence of template thickness on localization accuracy is further discussed in detail in Section 3.4.

To enhance computational efficiency, a moving window is adopted to capture local regions of the waterfall plot containing the PIG signal, and image template matching is then applied to automatically identify the pattern locations of the PIG striking the weld seams.

## 3. Experimental Study

### 3.1. DVS System Parameters and Deployment

The DVS system parameters are shown in Table 1. Deployed at a station in Southwest China, it uses a redundant fiber in the existing communication cable for distributed sensing, eliminating the need for dedicated sensing fibers and reducing deployment costs. The terrain is complex, with pipelines and cables crossing roads, wheat fields, villages, tunnels, bridges, hillsides, and rivers. Crude oil, natural gas, and refined oil pipelines run in parallel along some sections, so a single fiber can serve multiple pipelines. While this enables unified monitoring, it also introduces more complex background noise, making weak PIG signal extraction challenging.

The tracked PIG operates in a crude oil pipeline 5–10 m away from the fiber—a typical long-distance, weak-signal scenario. Weld-strike vibrations travel through the pipe and soil with significant attenuation. Leveraging the high sensitivity of the direct-detection DVS and our signal processing algorithms, we can still reliably capture these weak soil-transmitted signals amid strong environmental noise, providing a solid foundation for PIG localization and tracking.

### 3.2. Signal Preprocessing

**Figure 3:**
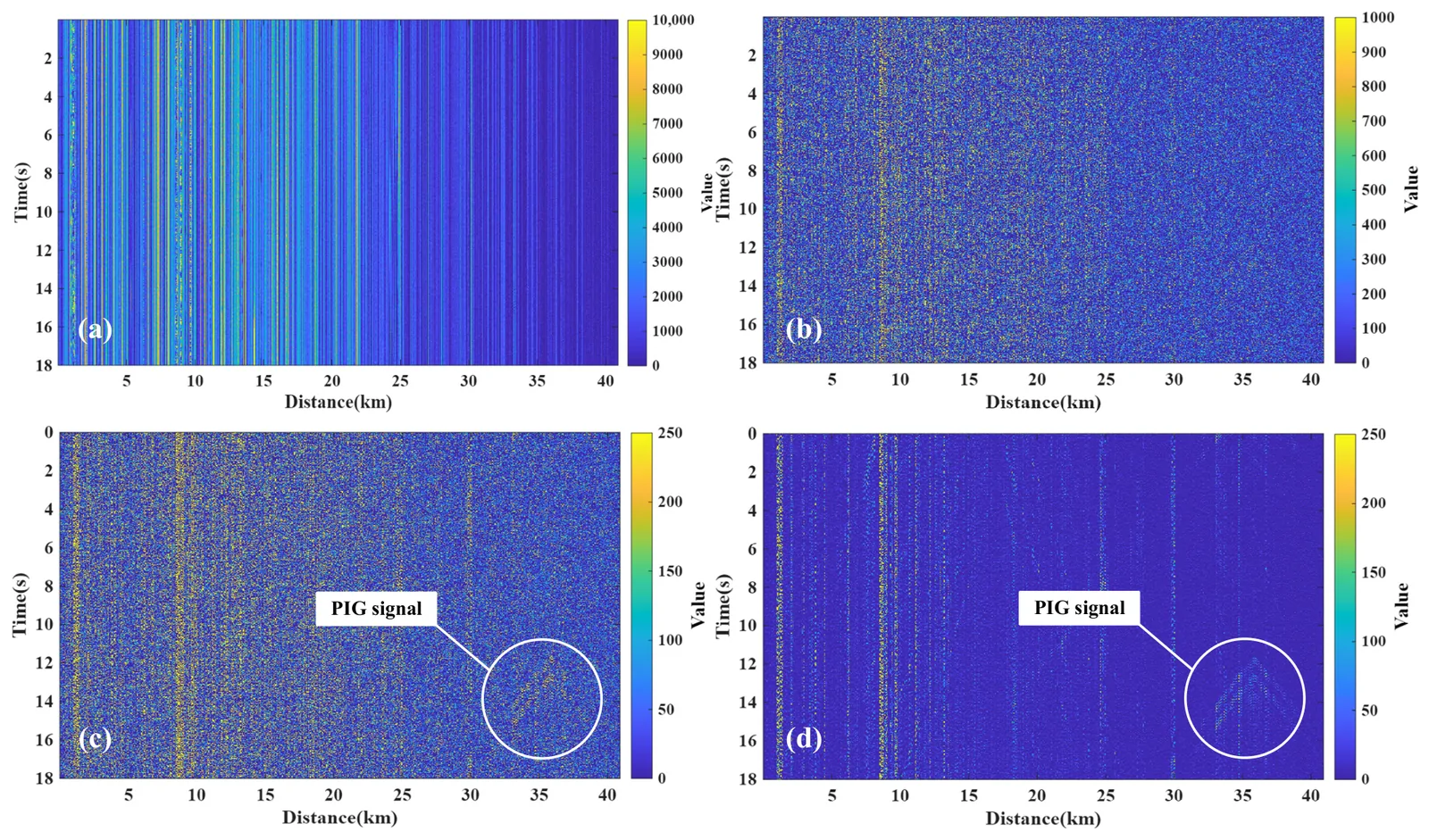
(**a**) Raw spatiotemporal waterfall plot. (**b**) Temporal differentiation with a 10-sample interval applied to (**a**). (**c**) Ten-sample temporal averaging followed by differentiation applied to (**a**). (**d**) Gaussian filtering of (**c**).

Figure 3a shows the spatiotemporal waterfall plot constructed from the raw Rayleigh backscattering signals acquired by a direct-detection DVS system. The alternating bright and dark speckle pattern originates from the constructive and destructive interference among a multitude of randomly distributed scattering centers within the fiber. The horizontal axis represents the distance along the fiber, and the vertical axis records the temporal evolution. To extract the weak vibration signatures induced by the passage of a PIG, temporal differentiation is commonly applied to the raw signals to highlight the vibration-induced variations in optical intensity. Figure 3b presents the result after temporal differentiation with a 10-sample interval (i.e., differencing with a lag of 10 time steps), where the background noise is extremely strong and the PIG-induced signal is almost completely submerged. Furthermore, by first averaging every ten consecutive temporal samples and then applying temporal differentiation, the waterfall plot shown in Figure 3c is obtained. Although the PIG signal becomes discernible, residual random noise and vertical-stripe interference remain significant. To address this, a Gaussian filter is employed to smooth the data, yielding the result in Figure 3d, where the vibration signal generated by the PIG is clearly enhanced and revealed. The persistent vertical-stripe noise in the waterfall plots primarily originates from systematic disturbances introduced by environmental background vibrations and other factors.

**Figure 4:**
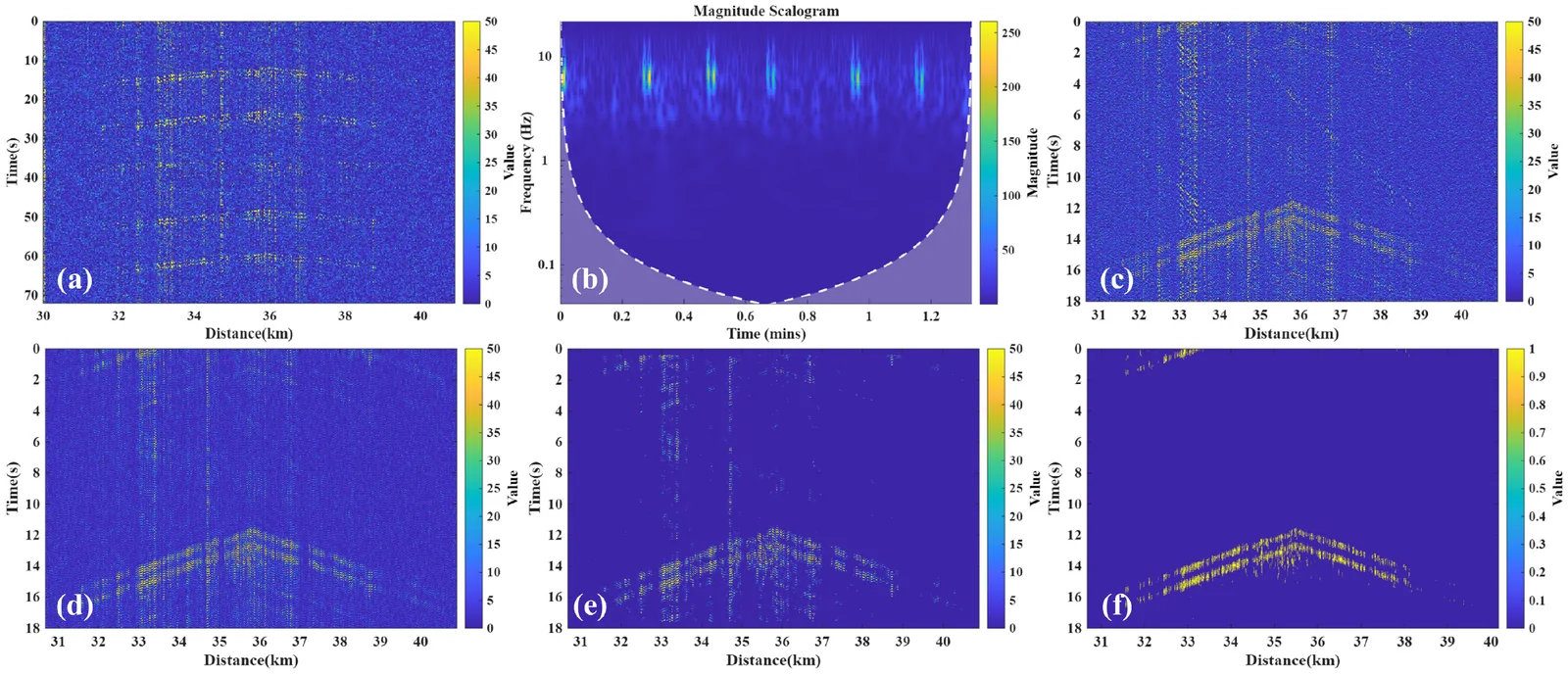
Time–frequency characteristics and extraction of PIG-induced weld-impact signals: (**a**) original spatiotemporal signal distribution; (**b**) wavelet time–frequency spectrum at 34.25 km; (**c**) original local waterfall plot; (**d**) result after 8–12 Hz wavelet filtering; (**e**) result after directional convolution filtering; (**f**) result after binarization.

In crude oil pipelines, adjacent welds are typically spaced approximately 12 m apart, corresponding to an impact interval of about 12 s at a PIG speed of 1 m/s (Figure 4a). Although the DVS spatial resolution is 20 m, successive weld-impact events can be distinguished through their temporal separation and V-shaped spatiotemporal signatures. The spatial sampling interval of 4.09 m corresponds to a theoretical quantization uncertainty of ±2.05 m, rather than the actual localization accuracy, which is also affected by signal quality and template parameters. Wavelet transform is performed on the time-domain signal at 34.25 km in Figure 4a, yielding the time–frequency spectrum shown in Figure 4b. From this spectrum, the vibration signal generated by the PIG impacting the pipeline weld can be identified, appearing as a specific frequency band with concentrated energy. During normal PIG operation, the vibration frequency characteristics excited by impacts on adjacent welds remain basically consistent along the entire crude oil pipeline. Continuous wavelet transform (CWT) filtering was performed on the preprocessed signals at a sampling frequency of 50 Hz using the analytic Morlet wavelet (amor). The wavelet scales were automatically determined using MATLAB R2025a’s default setting of 10 voices per octave. Wavelet coefficients outside the selected 5–8 Hz frequency band were set to zero, and the filtered signals were reconstructed using the inverse CWT. The resulting waterfall plot is shown in Figure 4d. Since the acoustic velocity of the PIG signal in the crude oil medium is relatively stable, its signal slope is related to the acoustic velocity. To further extract the signal, this paper introduces directional convolution filtering before binarization, with the relevant parameters listed in Table 2. This convolution kernel has directional band-pass characteristics, which can enhance linear structures along the V-arm direction while suppressing vertical stripe interference and random noise. After directional convolution filtering, the signal is binarized using the parameters in Table 3, yielding the final result shown in Figure 4f.

### 3.3. Signal Interpretation

**Figure 5:**
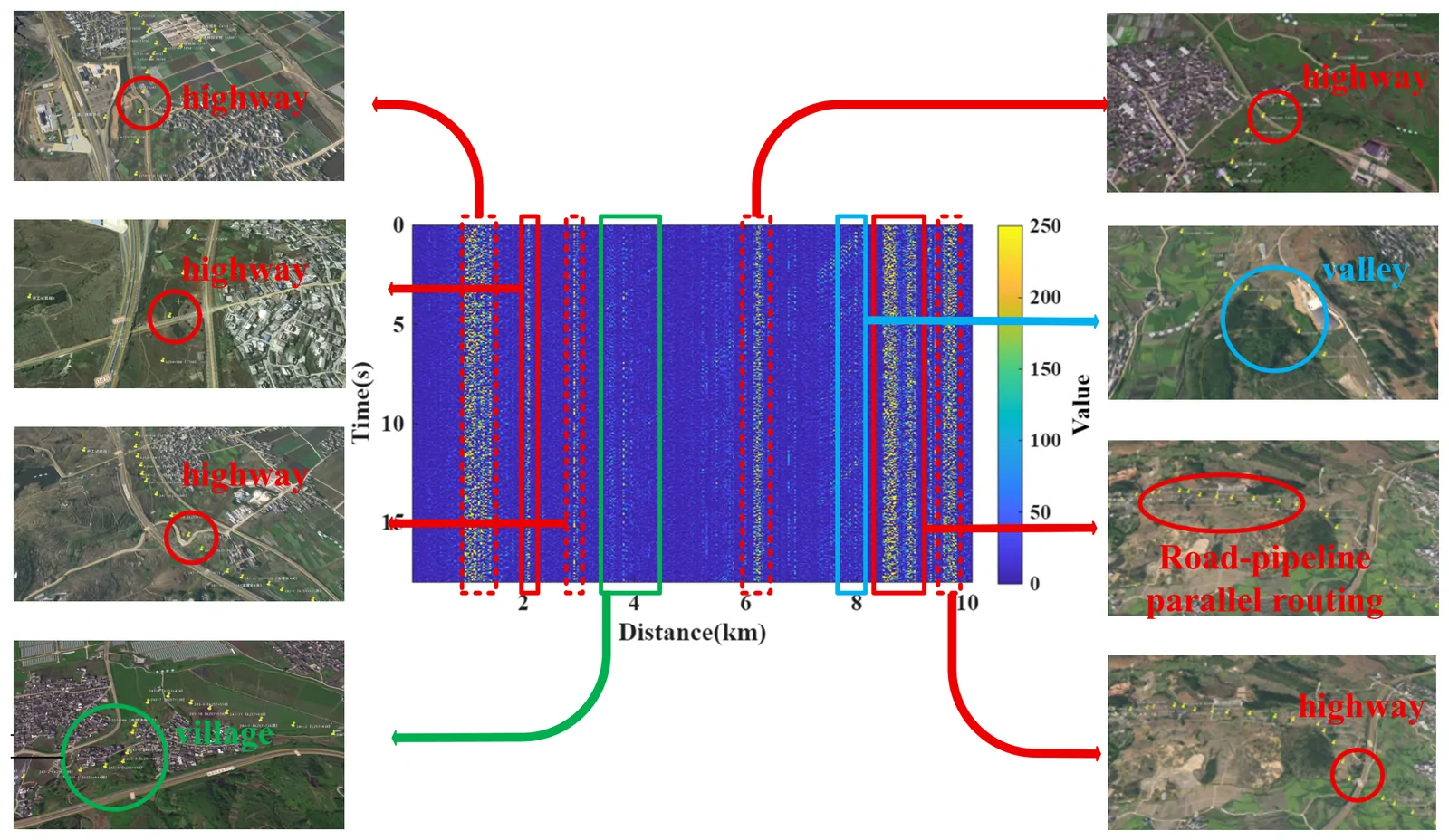
Noise signal sources in the first 10 km.

To identify the origin of the vertical-stripe noise observed in the first 10 km of the waterfall plot (Figure 5), we conducted a point-by-point comparison between the spatial distribution of the noise and the actual environmental features along the pipeline section. The results show that the strong vertical noise at 0.9–1.4 km, 2.1 km, 2.9 km, 6.2 km, and 9.7 km corresponds to locations where the pipeline runs parallel to or crosses highways; the interference at 3.5–4 km coincides with village human activities; and the noise at 7.9 km corresponds to micro-seismic activity in a valley gully. These noise patterns are spatially associated with nearby environmental features, suggesting that environmental vibrations may contribute to the observed disturbances.

**Figure 6:**
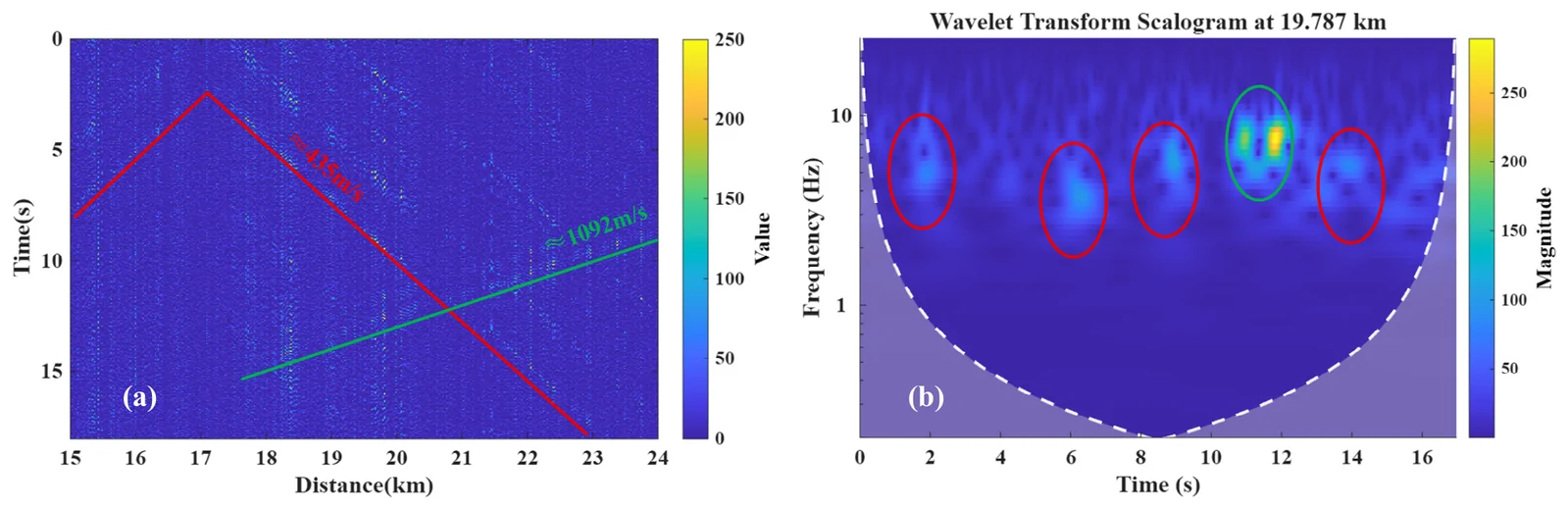
(**a**) Waterfall plot of the 15–24 km section; (**b**) Time–frequency spectrum at 19.787 km.

To investigate the capability of the DVS system to distinguish vibration signals from multiple pipelines, the 15–24 km section of Figure 3d is enlarged in Figure 6a, revealing two distinct propagation patterns. The red signal originates from an independent vibration source near 17 km in the natural gas pipeline, potentially associated with gas flow turbulence or valve throttling, with a measured propagation velocity of approximately 435 m/s. The green signal corresponds to PIG-induced weld impacts in the crude oil pipeline, with a measured propagation velocity of approximately 1092 m/s. These values are close to the reported acoustic velocities of 430.5 m/s for natural gas mixtures and 1095.7 m/s for heavy crude oil under specific thermodynamic conditions [21,22], with relative differences of approximately 1.05% and 0.34%, respectively. Although acoustic velocities depend on fluid properties and operating conditions, this agreement supports the physical interpretation of the two propagation patterns and their discrimination based on propagation velocity.

Furthermore, wavelet time–frequency analysis of the signal at 19.787 km (Figure 6b) reveals distinct energy concentration bands for the PIG-induced weld impacts (green circle) and the independent vibration source in the natural gas pipeline (red circle). These differences in spectral characteristics provide a basis for separating and extracting signals from different pipelines, demonstrating the potential of a single optical fiber for multi-source vibration monitoring.

**Figure 7:**
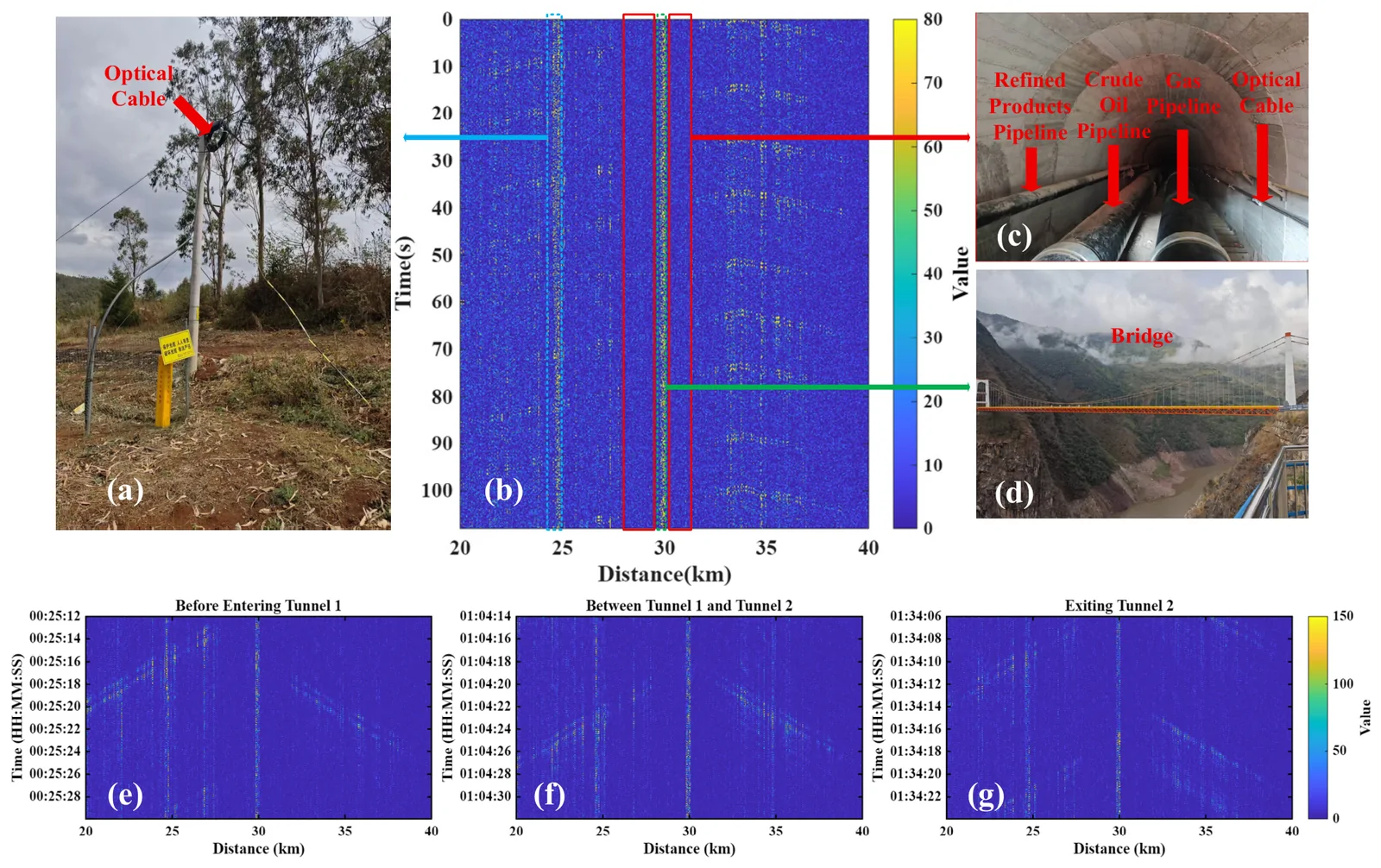
(**a**) Overhead installation of optical cable, with the yellow sign serving as a public awareness notice for optical cable protection; (**b**) Waterfall plot of the 20–40 km section; (**c**) optical cable installation inside the tunnel; (**d**) Bridge section. (**e**) Before the PIG enters Tunnel 1; (**f**) The PIG passing over the bridge between the two tunnels; (**g**) The PIG exiting Tunnel 2.

Figure 7 presents the waterfall plot containing PIG signals from 20 to 40 km. A persistent vibration signal is present at 24 km, attributed to the overhead installation of the optical cable rather than burial, with the signal primarily caused by environmental noise. Two signal absence zones appear before and after 29 km, corresponding to sections where the pipeline and optical cable traverse tunnels; the optical cable is suspended in steel pipes along the tunnel wall, making it difficult for vibrations to couple into the optical fiber. At the 29 km location, a bridge section generates continuous vibration, forming a persistent background signal. This indicates that the installation method of the optical cable directly affects the acquisition of target signals.

### 3.4. Method Validation

To achieve continuous PIG tracking, the localization method described in Section 2.2 was applied to the preprocessed signals obtained in Section 3.2. By extracting the characteristic signals generated by PIG–weld impacts through image processing, the method enables PIG localization despite background noise and sensing interruptions in the tunnel section. To improve detection reliability, template-matching thresholds and inter-frame horizontal coordinate constraints were employed to eliminate unreliable detections. Consequently, 583 valid detections were retained from 655 processed images, corresponding to a valid-result retention rate of 89.01%.

**Figure 8:**
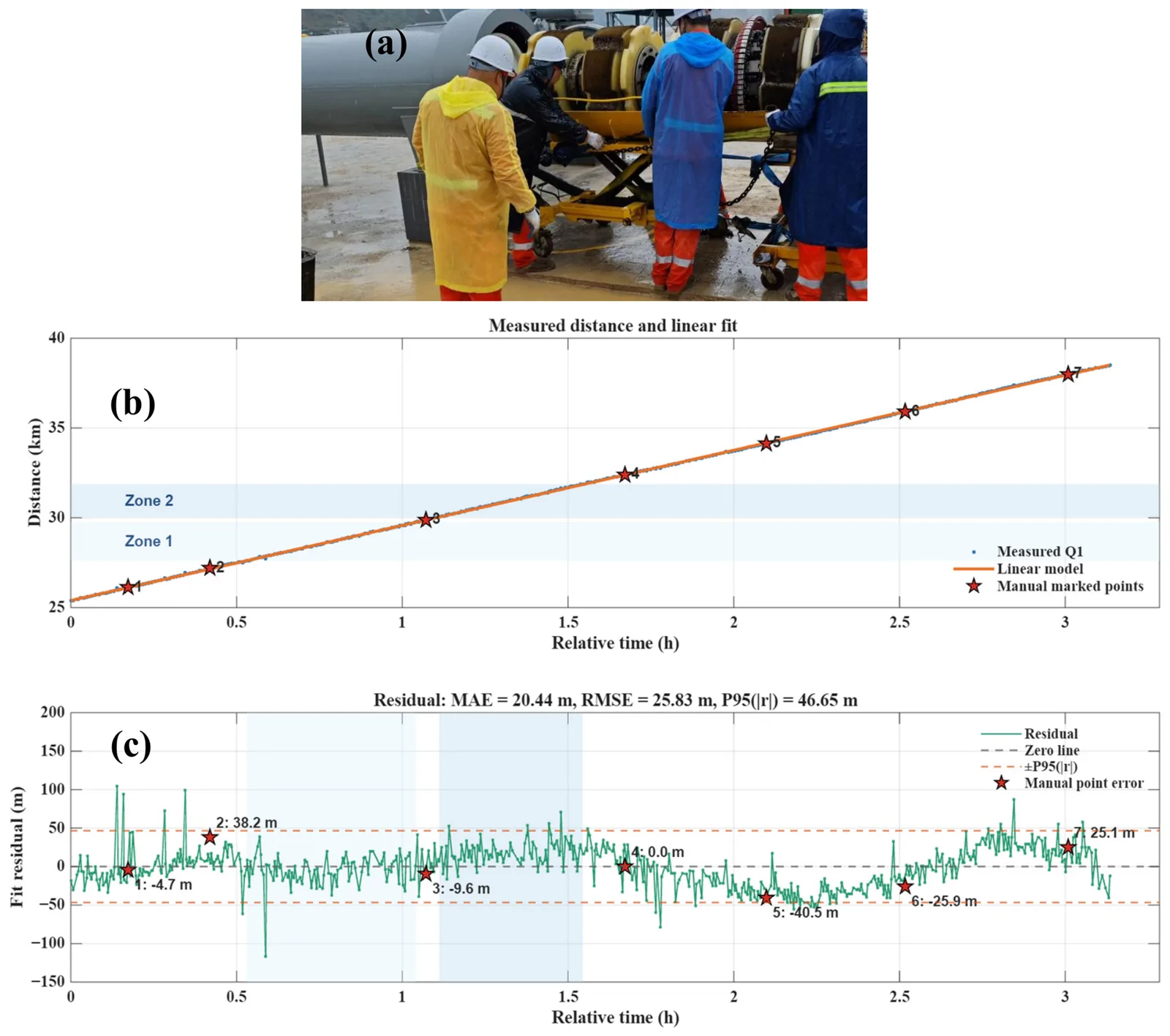
Field validation of PIG tracking using communication optical fibers: (**a**) PIG launching operation at the experimental site; (**b**) estimated PIG trajectory, linear fit, and manually recorded reference positions; (**c**) fitting residuals and localization errors at the manually recorded positions.

Figure 8 presents the field experiment and tracking results. The blue-shaded regions in Figure 8b,c indicate the tunnel section along the pipeline. The estimated PIG trajectory exhibits an approximately linear relationship between travel distance and time, consistent with the expected motion characteristics. To evaluate localization accuracy, the fitted trajectory was compared with seven manually recorded reference positions, yielding a mean absolute error (MAE) of 20.58 m, a root mean square error (RMSE) of 25.40 m, a maximum absolute error of 40.48 m, and a standard deviation of 27.30 m. Furthermore, residual analysis of the linear fit based on 583 valid detections yielded an MAE of 20.44 m, an RMSE of 25.83 m, and a 95th-percentile absolute residual of 46.65 m. These results demonstrate the consistency of the estimated trajectory with field observations and highlight the potential of existing communication optical fibers for practical PIG tracking without additional dedicated sensing hardware.

**Figure 9:**
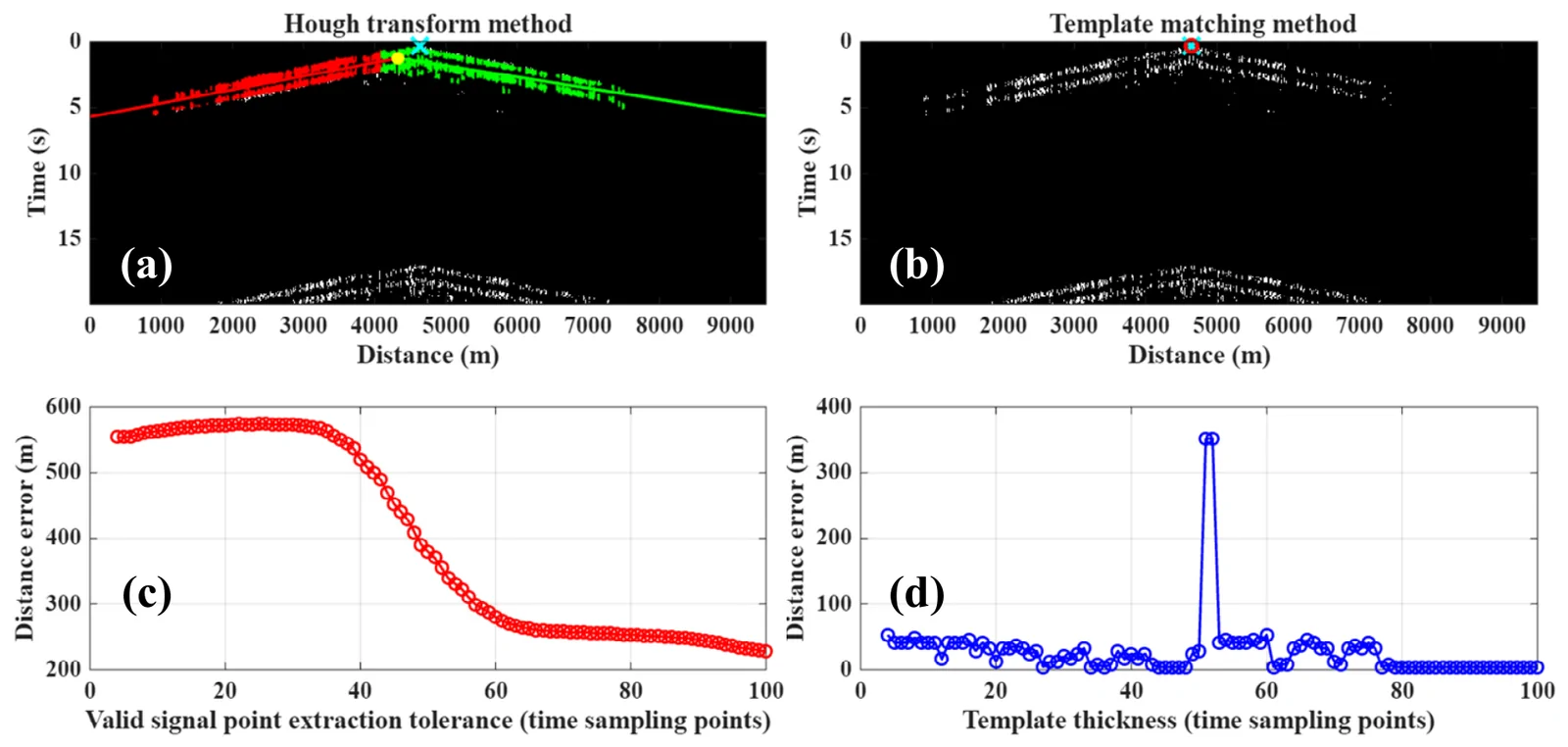
Comparison of localization accuracy and parameter sensitivity. (**a**) Hough transform results, where red and green points represent the valid points used to fit the two inclined lines, respectively; (**b**) Template-matching results; (**c**) Effect of extraction tolerance on localization error; (**d**) Effect of template thickness on localization error.

To quantitatively evaluate the proposed method, its localization performance was compared with that of a conventional Hough-transform-based method, using manually annotated apex positions of the V-shaped signals as references. As the Hough tolerance increased, the localization error decreased from approximately 550 m to 230 m. In contrast, the proposed template-matching method achieved errors below 50 m for most tested template thicknesses, except for a pronounced error peak near 52 sampling points (Figure 9). These results demonstrate higher localization accuracy of the proposed method on the tested dataset, while also revealing its sensitivity to template thickness.

## 4. Discussion

Wavelet filtering and template matching enable the localization of PIG-induced weld-impact events under environmental interference. In tunnel sections with reduced fiber coupling, localization relies on detectable acoustic waves propagating to adjacent sensing regions rather than extrapolation from previous positions. When no usable acoustic signatures are detected, reliable localization cannot be achieved. Furthermore, localization errors in these sections may be affected by optical cable redundancy and signal attenuation. Therefore, continuous PIG localization within signal-blind tunnel sections cannot be guaranteed and requires further quantitative evaluation. The separation between the pipeline and optical fiber affects vibration coupling and signal attenuation, potentially influencing weld-impact detection and localization performance. However, the direct-detection DVS system measures relative optical intensity variations rather than calibrated vibration amplitudes, making quantitative amplitude assessment difficult. The present field test demonstrates event localization at pipeline–fiber separations of 5–10 m, while the quantitative dependence of localization accuracy on separation distance requires further investigation.

The current template is constructed using a nominal PIG velocity of approximately 1 m/s. Variations in PIG velocity may alter the temporal characteristics of weld-impact signals and affect the correlation response and localization accuracy. Although Figure 9 evaluates the sensitivity to template thickness, the influence of PIG velocity variations requires further quantitative investigation.

The distinct propagation velocities and spectral characteristics observed in Figure 6 demonstrate the potential for distinguishing vibration sources from multiple parallel pipelines using a single optical fiber.

Although strong environmental noise was observed within the first 10 km, no PIG-induced weld-impact events were recorded in this section during the field test. Therefore, the localization performance of the proposed method under such severe noise conditions has not been experimentally verified and requires further investigation.

## 5. Conclusions

This study presents a physics-driven, training-free template-matching method for identifying and localizing PIG-induced weld-impact events using direct-detection DVS. Field experiments yielded 583 valid detections from 655 processed images, and the fitted trajectory achieved a mean absolute error of 20.58 m relative to seven manually recorded reference positions. Compared with the conventional Hough transform, the proposed method demonstrated lower localization errors under most tested template parameters. The method utilizes existing communication optical fibers without dedicated sensing cables. Future work will focus on adaptive template optimization and improving localization reliability under variable PIG speeds and signal-blind conditions.

## Figures and Tables

**Table 1:** DVS System Parameters.

Parameter	Value
Sensing distance	40 km
Spatial resolution	20 m
Maximum Measurable Frequency	250 Hz
Spatial sampling interval	4.09 m
Data matrix dimensions	10,000 × 10,000
Data time duration	20 s
Data spatial distance	40.9 km

**Table 2:** Directional Filter Parameters.

Core Parameters	Setting Value
Convolution kernel size	45 × 45
Standard deviation parallel to the line direction σ*∥*	13 pixels
Standard deviation perpendicular to the line direction σ⊥	1.8 pixels
Orientation angle θ	±14°

**Table 3:** Image Processing Parameters.

Core Parameters	Setting Value
Image enhancement algorithm	CLAHE
Local grid size	8 × 8
Contrast limit coefficient (ClipLimit)	0.01
Number of histogram bins	256
Threshold segmentation algorithm	Otsu
Threshold adjustment coefficient	1.0

## Data Availability

Data underlying the results presented in this paper are not publicly available at this time but may be obtained from the authors upon reasonable request.

## Abbreviations

The following abbreviations are used in this manuscript:

PIG	Pipeline Inspection Gauge
DVS	Distributed Vibration Sensing
Φ-OTDR	Phase-sensitive Optical Time-Domain Reflectometry
AOM	Acousto-Optic Modulator
EDFA	Erbium-Doped Fiber Amplifier
Cir	Circulator
FUT	Fiber Under Test
PD	Photodetector
FFT	Fast Fourier Transform
DAS	Distributed Acoustic Sensing
YOLO	You Only Look Once
